# Bi-dimensional principal gene feature selection from big gene expression data

**DOI:** 10.1371/journal.pone.0278583

**Published:** 2022-12-07

**Authors:** Xiaoqian Hou, Jingyu Hou, Guangyan Huang

**Affiliations:** School of Information Technology, Deakin University, Melbourne, Victoria, Australia; Chuo University, JAPAN

## Abstract

Gene expression sample data, which usually contains massive expression profiles of genes, is commonly used for disease related gene analysis. The selection of relevant genes from huge amount of genes is always a fundamental process in applications of gene expression data. As more and more genes have been detected, the size of gene expression data becomes larger and larger; this challenges the computing efficiency for extracting the relevant and important genes from gene expression data. In this paper, we provide a novel Bi-dimensional Principal Feature Selection (BPFS) method for efficiently extracting critical genes from big gene expression data. It applies the principal component analysis (PCA) method on sample and gene domains successively, aiming at extracting the relevant gene features and reducing redundancies while losing less information. The experimental results on four real-world cancer gene expression datasets show that the proposed BPFS method greatly reduces the data size and achieves a nearly double processing speed compared to the counterpart methods, while maintaining better accuracy and effectiveness.

## Introduction

Gene expression data contains the monitored expression levels of massive genes across different samples. With the rapid development of bioinformatics and data analytics, over 60 thousand genes can be identified with their expression profiles, and the use of gene expression data has been greatly promoted. Typical examples include identifying the genes that are related to a disease [1, 2] and enhancing the analysis of diseases and organisms at gene level [3], gene regulatory network inference [4], disease outcome classification [5], and cancer sub-types classification [6]. Due to the high cost of the experiments for obtaining the gene expression data, the number of patients, *n* (usually around 1,000), is much less than the number of genes, *p* (mostly over tens of thousand). This “*n* ≪*p*” property is called “the curse of dimensionality”, which challenges the use of gene expression data and the selection of important genes from thousands of detected genes [7]. As more and more genes can be detected and contained in the big gene expression data, precisely selecting the relevant and informative genes becomes more challenging as more redundancies are introduced at the same time.

In this paper, we provide a novel Bi-dimensional Principal Feature Selection (BPFS) method for efficiently extracting critical genes from big gene expression data. It applies the principal component analysis (PCA) method on sample and gene domains successively for extracting the relevant gene features and reducing redundancies, while losing less information. PCA [8, 9] is one of the most commonly used dimension reduction methods. It can remove high correlated variables without loss of too much information by performing orthogonal linear transformation on the feature domain to a new coordinate space with lower dimensions and independent features (using the principal components), since the top several principal components can keep the majority variation of the data. Therefore, the redundancy can be reduced from the highly correlated genes in the feature gene set [10] and the informative genes/features can be retained. PCA is widely used and studied, since it is a very effective technique for dimension reduction [11, 12], and the visualized information obtained from PCA can be used for further analysis on gene relationships.

However, classic PCA has limitation when applied directly to large number of genes/features due to the heavy computation of covariance. In [13], PCA is applied on the data matrix (i.e., genes on rows and samples on columns), but when the number of genes is large, the computational cost is extremely high due to the calculation of covariance matrix; this limitation is solved in [14] by avoiding the computation of principal components.

Similar to [14], our BPFS method does not use the principal components as new variables but extract the important original features in the process of constructing principal components. However, we keep the fundamentals of PCA and the usefulness of principal components and apply PCA on both the row domain and the column domain. So, in the BPFS method, our first PCA is used to reduce the features on columns and accordingly the gene size of the large gene expression data is reduced; this PCA process is very fast, because the gene expression dataset has only hundreds of samples and the computational cost of calculating the covariance is relatively low.

The features/genes with high contribution to the top *k* principal components are selected in the first PCA process; this reduces the size of the raw dataset. Then, our second PCA is applied on the filtered dataset that consists of selected genes and their expressions over the samples. The second PCA can be applied on genes, as the feature size has been reduced by the first PCA process. So, the second PCA is actually a classic PCA that can be easily adopted on the features/genes directly without exceeding the computational capacity. In the second PCA, the genes which contribute more to the computation of principal components are further selected. After the second PCA, the feature domain is filtered, the redundancy in the raw dataset is further reduced, and the remaining genes can be regarded as relevant and informative genes.

In contrast with the existing feature selection methods, the major differences and advantages of our BPFS method are threefolds, (i) we use PCA twice and the first on samples domain and the second on genes domain to overcome the computational capacity problem of classic PCA when it applies on large feature set; (ii) our method can also be used as a data cleaning step on the gene expression dataset to remove irrelevant genes and increase accuracy on feature selection without loss of much information; and (iii) the cleaned dataset can be further used for downstream analysis at gene level, such as cancerous genes relationship network construction, as this new dataset has less noise and redundancy.

In summary, this paper has the following contributions: (1) we provide a novel BPFS method for precise (efficient and accurate) gene feature selection, which can process big gene expression data. (2) we demonstrate on two real world cancer datasets that our BPFS method greatly reduces the data size and achieves a nearly double processing speed as the counterpart methods, while maintaining the same or even better accuracy. (3) we also show the effectiveness of our BPFS method, that is, the top two selected genes by our BPFS method can separate the normal samples and the samples with cancer very nicely.

The structure of this paper is as follows. In Section 2, we present the related work. In Section 3, we briefly introduce classic PCA and then detail our proposed method. In Section 4, we report the experimental results with discussion. We conclude our work in Section 5.

## Related work

The existing approaches for gene feature selection can be categorized into three types: filter method, wrapper method and embedded method [15, 16].

Filter method is a kind of method that only relies on the structure of the dataset and is independent of models and predictions [17]. For example, Ding et al. [10] proposed a minimum redundancy maximum relevance (mRMR) method to select feature genes from microarray datasets based on the relevancy and redundancy between variables or genes (such as minimize paired Euclidean distance between genes); Le et al. [18] introduced STatistical Inference Relief (STIR) on the basis of Relief [19], an algorithm developed by Kira and Rendell in 1992, which uses a statistical method to select features according to the calculated statistical dependency.

Wrapper method uses learning algorithms in the selection process, such as evolutionary algorithm [20], genetic algorithm [21] and swarm intelligence algorithms [22]. On gene expression analysis and cancer classification task, the wrapper methods can achieve high accuracy but are sensitive to the classifiers and also highly computational cost [23, 24].

Embedded method is a combination of two or more feature selection methods. Kavitha et al. [13] applied PCA [8, 9] to microarray data to reduce the number of features and used support vector machine recursive feature elimination (SVM-RFE) [25] to rank the selected genes. Alomari et al. [26] combined mRMR [10] and bat-inspired swarm intelligence algorithm to select genes. Sun et al. [27] proposed a kernel-based feature selection method for microarray data. Huang et al. [28] proposed FCSVM-RFE algorithm which combined k-means clustering and SVM-RFE [25] ranking method to select feature genes from microarray data. Recently, Al-Rajab et al. [29] proposed a feature selection method for colon cancer classification using information gain and genetic algorithm; Haque et al. [30] performed a mutual information based algorithm for feature selection from gene expression data.

Although these methods achieved great success on cancer related gene selection from small sized gene expression dataset, they do not work well with larger datasets due to computational capacity and complexity. Kavitha’s method [13] cannot be applied to process our datasets (containing 60,482 genes), since it cannot handle a large number of genes. FCSVM-RFE [28] suffers from high computational cost when applying k-means clustering on over 60 thousands genes. The number of feature genes selected by STIR [18] is usually very large and the selection process is very time-consuming.

Unlike FCSVM-RFE [28] and STIR [18], our BPFS method avoids direct pair-wise calculations on the gene domain, by firstly conducting operations on the sample domain to reduce the size of the gene domain, then conducting the gene domain operations.

## Method

In this section, we first introduce the procedure of PCA. Then we present our proposed BPFS method to select informative genes and reduce data size by reducing the dimensionality.

### Preliminary knowledge of PCA

PCA is one of the most commonly used dimension reduction methods [9]. PCA can perform an orthogonal linear transformation on gene expression data to a new coordinate space with lower dimensions and features. The new features, which are called principal components (PC), are independent only if the features in raw data are jointly normally distributed. The first principal component has the greatest variance, the second PC has the second greatest variance and so on. PCA creates a new feature domain and reaches the aim of reducing dimensionality by computing principal components. Steps of PCA are as follows:

Let *M* be the input matrix. The dimension of *M* is *m* × *n*, *m* is the number of samples/observations, *n* is the number of features/attributes.

- Calculate the mean value of each column of matrix *M* by Eq (1)
  $$\begin{array}{c} \bar{X}=\frac{1}{m}\sum_{i=1}^{m}X_{i}. \end{array}$$ (1)
  *X*_*i*_ is the vector of observation values for sample *i* with dimension 1 × *n*.
- Subtract the mean values $\bar{X}$ from the input matrix *M* and compute the covariance matrix *C* of the modified data by Eq (2)
  $$\begin{array}{c} C=\frac{1}{m-1}\sum_{i=1}^{m}{\left(X_{i}-\bar{X}\right)}^{T}\left(X_{i}-\bar{X}\right). \end{array}$$ (2)
  $\bar{X}$ is the vector of mean values calculated in (1).
- Calculate the eigenvalues λ_*n*_ and the eigenvectors *v*_*n*_ of the covariance matrix *C* by solving Eq (3)
  $$\begin{array}{c} Cv_{n}=\lambda_{n}v_{n}. \end{array}$$ (3)
- Rank the eigenvalues from high to low.
- Pick the top *k* eigenvalues and the corresponding eigenvectors. The number of eigenvalues is selected based on the cumulative proportion of variance (*PPV*). *PPV* and the cumulative *PPV*, i.e., cPPV, are calculated by Eqs (4) and (5) respectively,
  $$\begin{array}{c} PPV_{i}=\frac{\lambda_{i}}{\sum_{j=1}^{n}\lambda_{j}}, \end{array}$$ (4)
  $$\begin{array}{c} cPPV_{i}=\sum_{j=1}^{i}PPV_{j}. \end{array}$$ (5)
- Generate the new dataset by projecting each row of matrix *M* to a *k*-dimensional space created by the *k* eigenvectors, where *k* < *n* and *k* = *min*{*i*|*cPPV*_*i*_ > = *α*}, *α* is a predefined threshold. The selected *k* eigenvectors are also called principal components (PCs).

We can use many existing open-source functions to implement PCA on a dataset, such as the *prcomp* function in R [31], which is based on Singular Value Decomposition (SVD) [32] of the data matrix.

### The proposed Bi-dimensional principal feature selection (BPFS) method

Our method is inspired by Kavitha’s method [13]. Although Kavitha’s method [13] can successfully reduce the dimension of small-size datasets (less than 10,000 genes), it barely works on large-size datasets due to the computational cost of classic PCA. To overcome this problem, we propose a novel BPFS method. The details of BPFS are stated in Algorithm 1.

**Algorithm 1** BPFS feature selection approach.

**Input:** The gene expression data matrix, *A*, with *m* samples and *n* genes; the threshold, *α*, for selecting PCs; the percentile threshold, *β*, for the loading score; the percentile threshold, *τ*, for the contribution score;

**Output:** The smaller gene expression data matrix, *C*, with *m* samples and *k* genes, *k* < *n*;

1: Perform PCA on matrix *A*;

2: Select top *α* PCs;

3: Choose the genes with top *β* absolute loading scores on the selected PCs;

4: Filter the raw matrix *A* based on the selected genes and denote the filtered matrix as *B*^′^;

5: Transform matrix *B*^′^ with genes on rows and samples on columns and denote it as *B*;

6: Perform PCA on matrix *B*;

7: Select top *α* PCs;

8: Choose the genes with top *τ* contribution scores for each selected PC;

9: Filter matrix *B* based on the selected genes and denote the filtered matrix as *C*′, transform *C*′ with samples on rows and genes on columns and denote this transformed matrix as *C*;

10: **return** matrix *C*;

The input matrix for our method is a gene expression dataset, *A*, with *m* samples and *n* genes. The first four lines in Algorithm 1 belong to the first phase of our method. In line 1, we performed PCA on matrix *A* with samples on rows and genes on columns. The PCs are ranked based on their *PPV* and the top *α* will be selected, where *α* is a predefined threshold. The projection of each sample on *PC*_*i*_, denoted as $P_{PC_{i}}$ in Eq (6), can be written as a linear combination of column variables (genes for gene expression data) [12], where *A*_*j*_ is the column vector of input matrix *A*, *γ*_*i*_ is a row vector of (*γ*_*i*,1_, *γ*_*i*,2_, …, *γ*_*i*,*n*_). *γ*_*i*_ is called loading scores for *PC*_*i*_ and can be calculated by Eq (7)

$$\begin{array}{c} P_{PC_{i}}=A\gamma_{i}^{T}=\sum_{j=1}^{n}\gamma_{i,j}\times A_{j}, \end{array}$$ (6)

$$\begin{array}{c} \gamma_{i}^{T}=v_{i}\times \sqrt{\lambda_{i}}, \end{array}$$ (7)

where λ_*i*_ is the eigenvalue for *PC*_*i*_, *v*_*i*_ is the corresponding eigenvector. For instance, *γ*_*i*,1_ is the loading score of gene 1 on *PC*_*i*_.

A larger loading score means the corresponding variable (gene) has a stronger impact on the computation of the specific PC. We rank the loading scores with the corresponding genes for each selected PC and choose the top *β* genes. Then, we take the union of the selected genes from each selected PC as a feature (gene) subset obtained from the first phase of our approach.

Now we get a matrix with less genes from the original matrix, by filtering out those genes that are not in the gene subset generated from the first phase, as well as their corresponding expression values/columns in the original matrix, *A*. Then, we enter into the second phase of our method. Lines 5 to 9 in Algorithm 1 are for this phase. This time we conduct PCA on the columns (genes) domain. To do this, we transform the filtered matrix, *B*^′^, into the matrix with genes on rows and samples on columns and denote this transformed matrix as *B*. The PCs are selected based on the *cPPV* and the threshold, *α*, following the same process as in first phase. Then, we use the contribution score, *ctrib*_*g*,*i*_, of gene *g* on the selected, *PC*_*i*_ [33], to further filter the genes and reduce the dimensionality which is given by

$$\begin{array}{c} ctrib_{g,i}=\frac{f_{g,i}^{2}}{\sum_{g}f_{g,i}^{2}}=\frac{f_{g,i}^{2}}{\lambda_{i}}, \end{array}$$ (8)

where *f*_*g*,*i*_ is the factor score calculated by SVD [32] of the input matrix, and λ_*i*_ is the eigenvalue of *PC*_*i*_.

Specifically, let *B* be the input matrix with *m* samples and *y* genes. *B* is a *y* × *m* matrix and has a SVD,

$$\begin{array}{c} B=USV^{T}, \end{array}$$ (9)

where *U* is the *y* × *y* matrix containing the left singular vectors of *B*, *V* is the *m* × *m* matrix containing the right singular vectors of *B*, *S* is the *y* × *m* rectangular diagonal matrix with non-negative values, which are called the singular values of *B*. The values of *S* are the square roots of the positive eigenvalues of *BB*^*T*^ (*BB*^*T*^ can be considered proportional to the empirical sample covariance matrix of the dataset, *B* [34]). The factor score matrix, *F*, is defined by *F* = *US*, which is a *y* × *m* matrix. *f*_*g*,*i*_ is the (*g*, *i*) entry of *F*.

We keep the genes with the top *τ* contribution scores for each selected PC and take the union of genes from each PC. After lines 1 to 9, the feature (gene) domain is filtered twice while the number of samples is unchanged, and the final matrix with selected feature genes is denoted as *C*.

## Experiments

In this section, we evaluate our method through experiments conducted on real-world gene expression datasets. We first introduce the experimental setup, including datasets, the counterpart methods and the evaluation metrics. We then evaluate the proposed method for cancer classification in terms of efficiency, accuracy and effectiveness.

### Experimental setup

#### Datasets

We test our approach on four representative cancer gene expression datasets from TCGA project [35]: Papillary Thyroid Carcinoma (THCA), Kidney Renal Clear Cell Carcinoma (KIRC), Primary Prostate Cancer (PRAD), and Lung Squamous Cell Carcinoma (LUSC). The datasets are publicly available and include ground truth labels (with or without cancer) for evaluation purpose.

The datasets are summarized in Table 1, including the number of detected genes, normal samples and samples with cancer. The gene expression levels in the datasets are normalized by TPM (transcripts per million) which is one of the most popular gene expression level normalization methods [36].

**Table 1:** Experimental datasets.

Dataset	Genes	Normal Samples	Samples with Cancer
THCA	56309	59	512
KIRC	56909	72	541
PRAD	56467	52	501
LUSC	56795	49	502

#### The counterpart methods

We compare our proposed method with the following state-of-the-art methods: FCSVM-RFE [28] and STIR [18], based on the performance on the cancer classification task.

- FCSVM-RFE [28] is an embedded method combining the clustering algorithm and SVM-RFE [25] feature ranking method. It first clusters the genes/features into groups and then selects the representative gene/feature for each group. Finally, it ranks the representative genes/features by SVM-RFE [25].
- STIR [18] is a filter method that based on the Relief family [19] but adding statistical significance to the features as thresholds to select the most important features.

**Fig 1:**
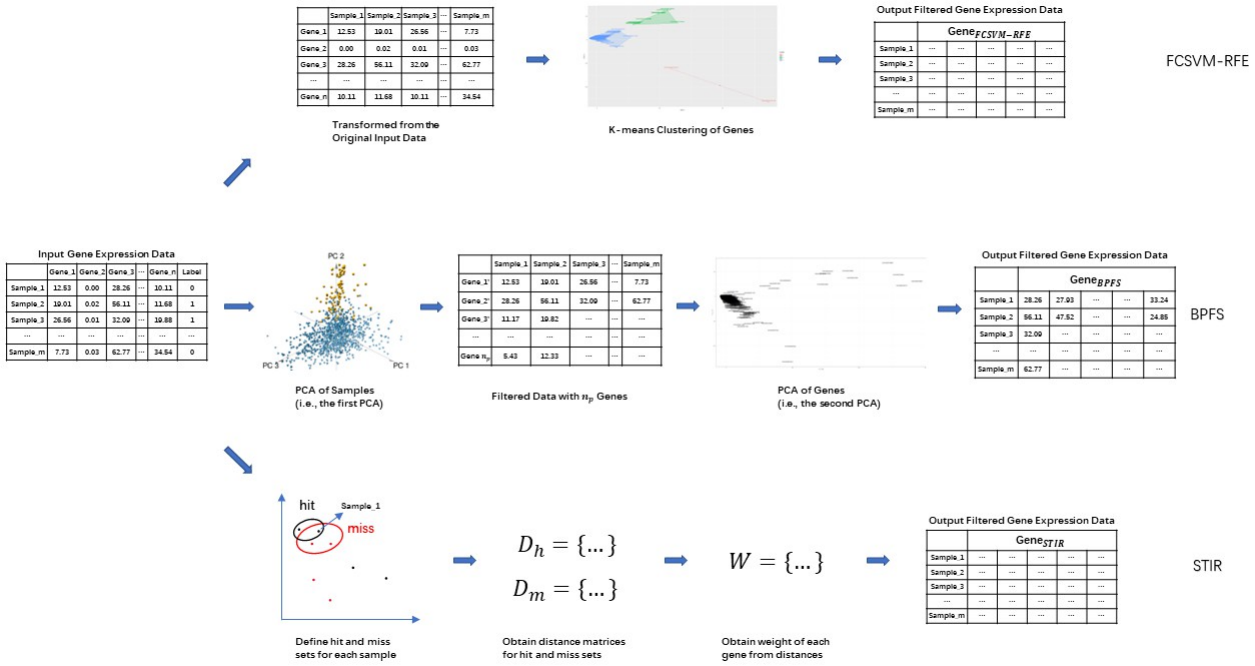
Different mechanisms on feature selection among FCSVM-RFE, STIR and our BPFS.

Fig 1 shows the different mechanism of FCSVM-RFE [28], STIR [18] and our method on feature selection. FCSVM-RFE [28] takes the transformed gene expression data matrix (with genes on rows and samples on columns) as input. Then it applies clustering method to cluster the genes into several groups. In [28], it runs parameter setting experiments on Leukemia dataset (with 72 samples and 7129 genes), the best number of clusters is 80, which is approximately 1% of the number of genes. From each cluster, the top 5 genes which are closest to the center of the cluster are chosen as representative genes. Then it applies a feature ranking algorithm to the chosen representative genes. The final selected gene domain is made up of the top ranked genes. STIR [18] takes the raw gene expression data matrix as input. It first defines the neighbourhood, the hit and miss sets for each sample. For example, the hit set for sample_1 contains the samples with the same label as sample_1, while the miss set contains the samples with different label as sample_1. Then it calculates the distance between sample_1 and the other samples in hit and miss set. Finally, a weight matrix is obtained from the calculated distance matrices, which leads to the final gene domain selection.

The mechanism of BPFS has been explained in detail in the Method Section. In brief, BPFS takes the raw gene expression data matrix as input. It applies PCA to the sample domain first, and chooses the genes with high contribution (based on the loading score) to the top PCs. As shown in Fig 1—PCA of Samples (i.e., the first PCA), the samples are clustered in the space of the selected top PCs (says, top 3), and the chosen genes are those with higher contribution (i.e., larger coefficient in the linear combination representation of the selected PCs), so they can better represent the selected PCs and can be used to distinguish cancerous and normal samples/patients. Additionally, by doing operations on the sample domain first, BPFS can reduce the computation time by avoiding heavy pair-wise calculations on the gene domain. Then, BPFS applies PCA to the filtered gene domain, and further select the genes with high contribution (based on the contribution score) to the top PCs. From the second phase of BPFS, as shown in Fig 1—PCA of Genes (i.e., the second PCA), the genes are clustered based on their expressions in the samples in the space of selected PCs (says, top 2 PCs). By looking at the contribution score of each gene at the top PCs, the selected genes (with higher contribution scores) are more important to decide the PCs. In the other words, the selected genes from the second phase can better distinguish the genes themselves. To summarize, as show in Fig 1, the first PCA keeps *n*_*p*_ genes (for example, Gene_1 and Gene_3) out of *n* genes from the input gene expression data, and the second PCA further selects genes (for example, Gene_2^′^) from the gene set obtained from the first PCA and get the final selected gene set *Gene*_*BPFS*_. By choosing genes with high contribution to decide the top PCs, BPFS keeps the major information from the raw data.

#### Evaluation metrics

We divided the raw data into training set and testing set. The training set contains 80% of the total number of rows of the raw data, while the testing set contains the rest 20% of the total rows. Then we applied BPFS, FCSVM-RFE [28] and STIR [18] separately on the training set to get the feature gene set and performed SVM classification on the filtered training set with selected genes. Finally we tested the performance on the testing set in terms of accuracy, sensitivity, specificity, precision, balanced accuracy and F1-score.

In the cancer classification task using SVM, we denote the patient with cancer as positive. Let *P* and *N* be the total number of positive cases and negative cases, respectively. Let *TP* and *FN* represent the number of correct and incorrect prediction for patients with cancer, respectively. *TN* and *FP* are similarly defined for patients without cancer. We focus on evaluating how correctly the patients are predicted and how correctly the patients with cancer can be identified, which are represented by accuracy and sensitivity, respectively,

$$\begin{array}{c} Accuracy=\frac{TP+TN}{P+N}, \end{array}$$ (10)

$$\begin{array}{c} Sensitivity=\frac{TP}{P}. \end{array}$$ (11)

In addition to accuracy and sensitivity, we also included metrics of specificity, precision, balanced accuracy and F1-score in our comparison evaluations.

$$\begin{array}{c} Specificity=\frac{TN}{N}, \end{array}$$ (12)

$$\begin{array}{c} Precision=\frac{TP}{TP+FP}, \end{array}$$ (13)

$$\begin{array}{c} BalancedAccuracy=\frac{Sensitivity+Specificity}{2}, \end{array}$$ (14)

$$\begin{array}{c} F1=\frac{2TP}{2TP+FP+FN}. \end{array}$$ (15)

The values of the evaluation metrics are ranged from 0 to 1 and the higher value represents the better performance.

### Performance evaluation

#### Optimal parameter setting

**Fig 2:**
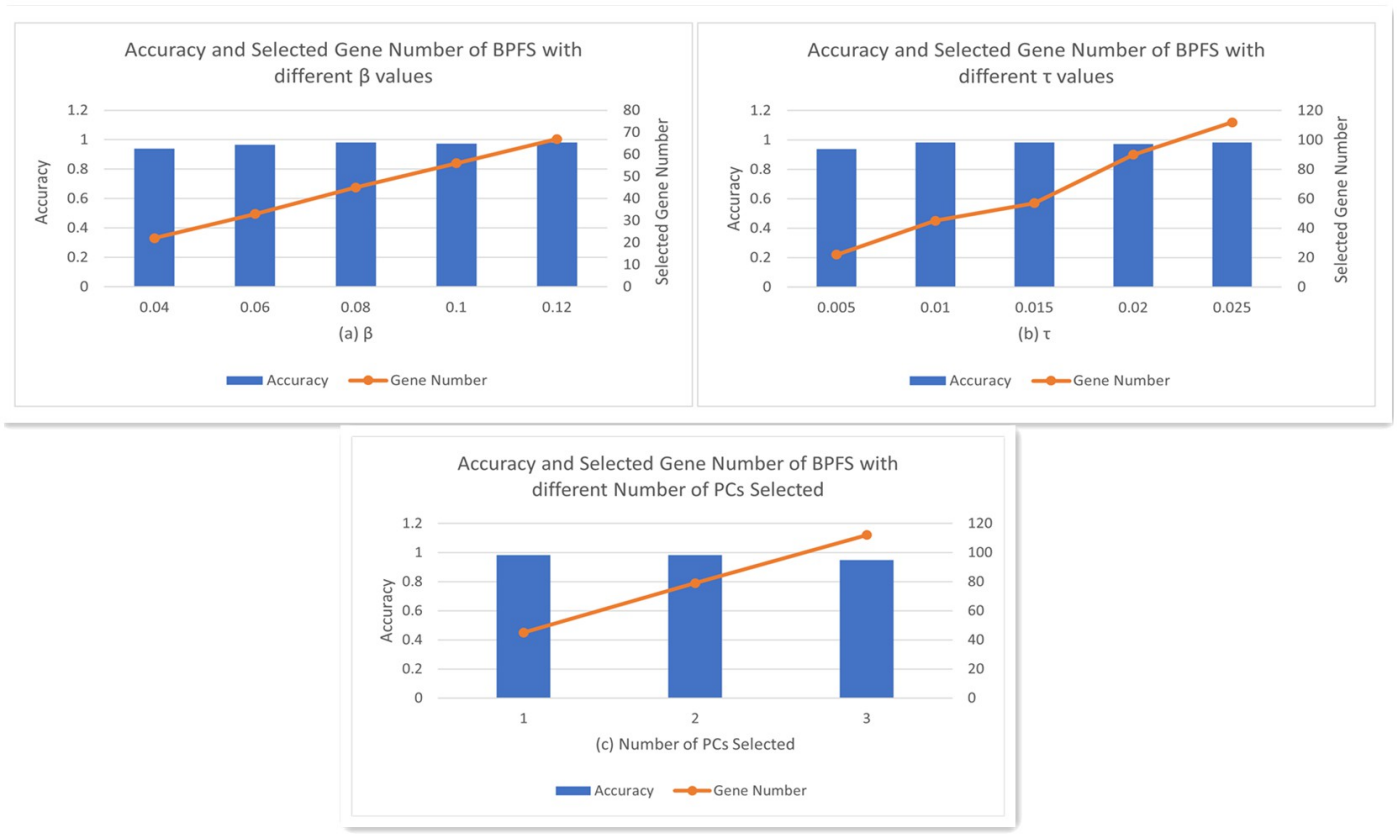
Accuracy vs different parameter values on THCA datasets.

In our algorithm, we have three parameters, which are *α* *β* and *τ*. Firstly, we set *α* = 1, which means the first principal component will be selected in both first PCA and second PCA, as the first PC represents the maximum variance direction of the data and best approximates the data in the least squares sense. To determine the values for *β* and *τ*, we start with setting *τ* = 1%, the performances in accuracy on THCA dataset with different values of *β* are compared. As shown in Fig 2(a), the best accuracy (0.9825) occurs when *β* = 8%. Then we set *β* = 8% and compare the performances in accuracy on THCA dataset with different values of *τ*. As shown in Fig 2(b), the best accuracy (0.9825) occurs when *τ* = 1%. Additionally, Fig 2(c) shows the performances with different number of PCs selected with fixed *β* = 8% and *τ* = 1%.

In the following experiments, we will keep *α* = 1, *β* = 8% and *τ* = 1%, and the associated best number of gene selected is 45. The parameters for FCSVM-RFE [28] and STIR [18] are set based on their original papers.

#### Accuracy

In Tables 2 to 5, we report the classification results of the counterparts and our proposed method on four cancer datasets: THCA, KIRC, PRAD, and LUSC. We bold the results of our method if it is not worse than any counterparts.

**Table 2:** Classification effectiveness analysis on the THCA cancer dataset.

	Accuracy	Sensitivity	Specificity	Precision	Balanced Accuracy	F1-score
FCSVM-RFE [28]	0.9737	0.8333	0.9902	0.9091	0.9118	0.8696
STIR [18]	0.9737	0.75	1	1	0.875	0.8571
**BPFS (proposed)**	**0.9825**	**0.8333**	**1**	**1**	**0.9167**	**0.9091**

**Table 3:** Classification effectiveness analysis on the KIRC cancer dataset.

	Accuracy	Sensitivity	Specificity	Precision	Balanced Accuracy	F1-score
FCSVM-RFE [28]	0.9918	1	0.9907	0.9333	0.9954	0.9655
STIR [18]	0.9836	0.9286	0.9907	0.9286	0.9597	0.9296
**BPFS (proposed)**	**1**	**1**	**1**	**1**	**1**	**1**

**Table 4:** Classification effectiveness analysis on the PRAD cancer dataset.

	Accuracy	Sensitivity	Specificity	Precision	Balanced Accuracy	F1-score
FCSVM-RFE [28]	0.9364	0.2222	1	1	0.6111	0.3636
STIR [18]	0.9455	0.44444	0.9901	0.8	0.7173	0.5714
**BPFS (proposed)**	**0.9727**	**0.6667**	**1**	**1**	**0.8333**	**0.8**

**Table 5:** Classification effectiveness analysis on the LUSC cancer dataset.

	Accuracy	Sensitivity	Specificity	Precision	Balanced Accuracy	F1-score
FCSVM-RFE [28]	1	1	1	1	1	1
STIR [18]	0.9909	1	0.9904	0.8571	0.9952	0.9231
**BPFS (proposed)**	**1**	**1**	**1**	**1**	**1**	**1**

For THCA, as shown in Table 2, all three methods have good performance, while BPFS performs the best (0.9825, 0.8333, 1, 1, 0.9167, 0.091 for six evaluation metrics respectively). For KIRC and LUSC datasets, as shown in Tables 3 and 5, BPFS achieves 100% for all six evaluation metrics, which means that the gene subsets obtained by our method can be used to correctly classify the normal samples and samples with cancer. STIR [18] performs slightly worse than BPFS in both datasets, while FCSVM-RFE [28] performs slightly worse than BPFS on KIRC and same on LUSC. For PRAD data, as shown in Table 4, BPFS achieves the best performance (0.9727, 0.6667, 1, 1, 0.8333, 0.8 for six evaluation metrics respectively), which is 3% better than STIR [18] and 4% better than FCSVM-RFE [28] in accuracy.

Overall, our method achieves the best result on all four datasets, as firstly, BPFS keeps the first principal component in the first phase PCA, which ensure we do not lose much information during the PCA process.

#### Efficiency

**Fig 3:**
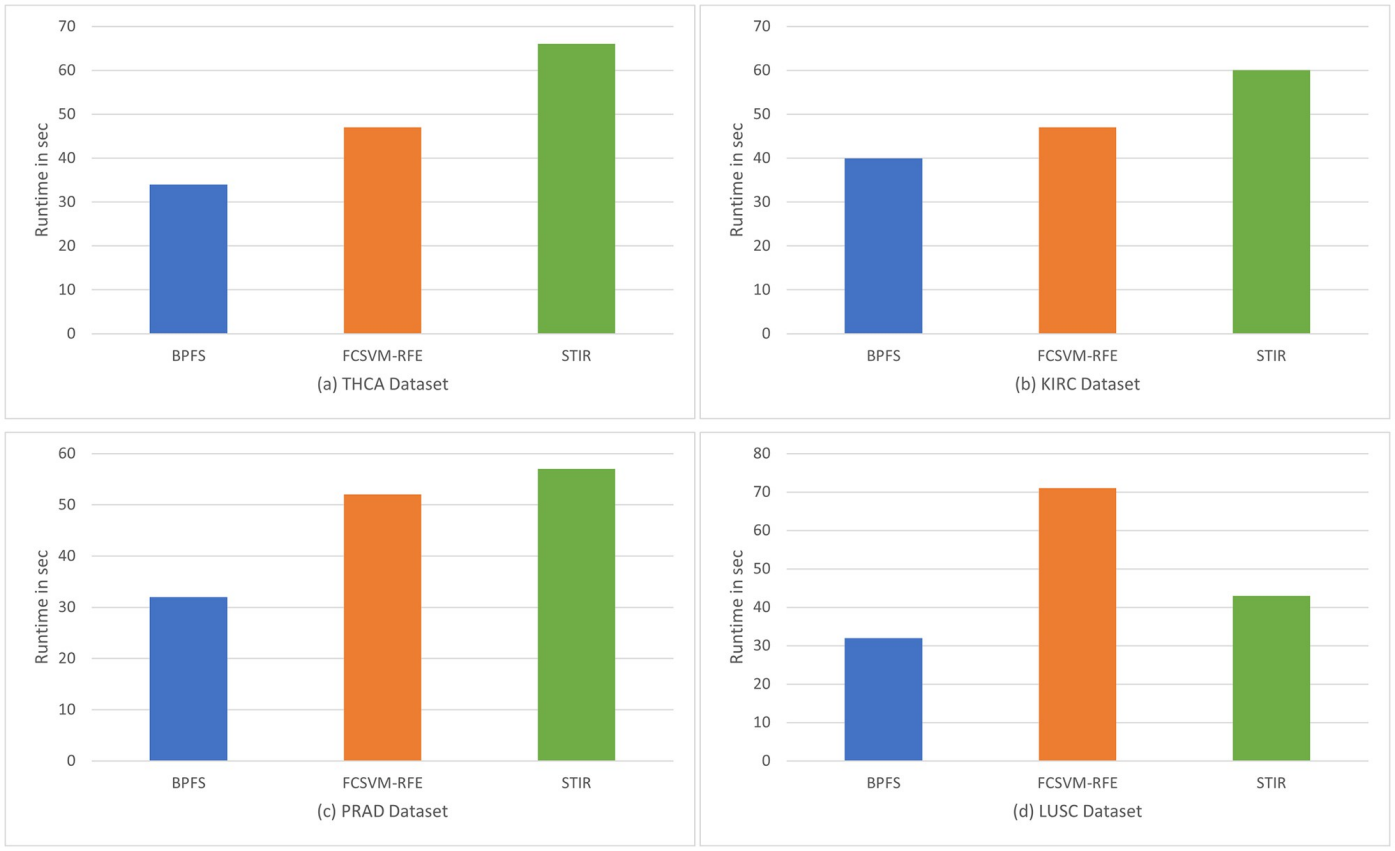
Runtime comparisons on four datasets.

Fig 3 shows the runtime of FCSVM-RFE [28], STIR [18] and BPFS in seconds. For THCA data, as shown in Fig 3(a), FCSVM-RFE [28] takes 47 seconds, STIR [18] takes 66 seconds and BPFS needs 34 seconds, which is 25% faster than FCSVM-RFE [28] and nearly doubles STIR’s speed. For KIRC data, as shown in Fig 3(b), FCSVM-RFE [28] takes 47 seconds, STIR [18] takes 1 minute and BPFS takes 40 seconds. For PRAD data, as shown in Fig 3(c), FCSVM-RFE [28] takes 52 seconds, STIR [18] takes 57 seconds and BPFS takes 32 seconds. For LUSC data, as shown in Fig 3(d), FCSVM-RFE [28] takes 71 seconds, STIR [18] takes 43 seconds and BPFS only takes 32 seconds. For all four datasets, our method has the fastest computational speed, as the first phase PCA does not require pair-wise calculation of the gene domain but reduces the size of genes for the second phase gene domain PCA, which potentially reduces the computational time.

#### Effectiveness

In Table 6, we report the number of genes selected by BPFS, FCSVM-RFE [28], STIR [18]. From the data size perspective, Table 6 and the previous exprimental results show that BPFS can successfully reduce the gene domain size from over 50,000 to 45.

**Table 6:** Number of genes selected.

	THCA	KIRC	PRAD	LUSC
No. of genes selected by BPFS	45	45	45	45
No. of genes selected by FCSVM-RFE	80	80	80	80
No. of genes selected by STIR	11915	8681	9648	6079

#### Ablation study

In this part, we will show the importance of the second phase of PCA by comparing the performance of BPFS with the method which only has the first PCA on the sample domain and select genes with a smaller *β*.

**Fig 4:**
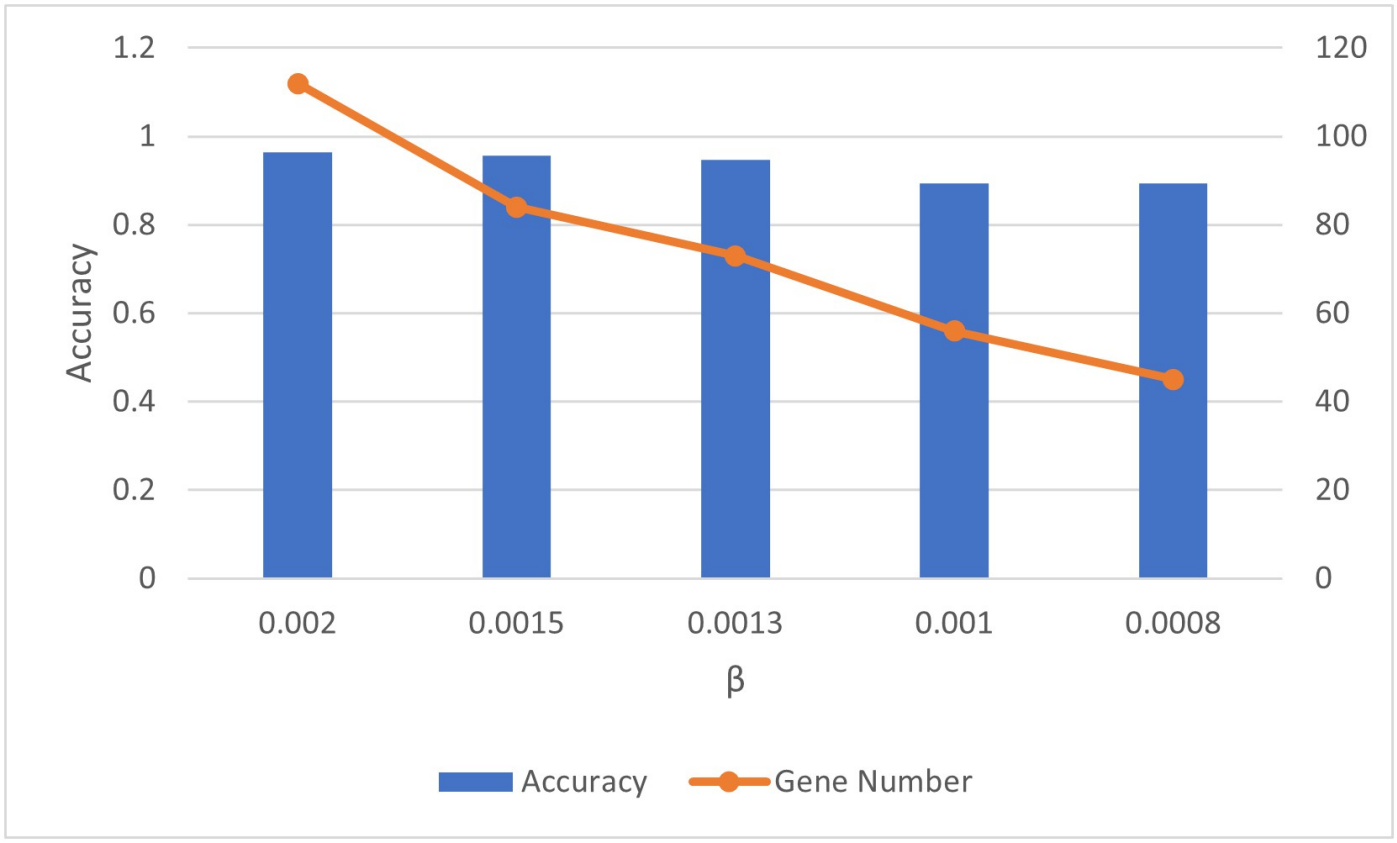
Accuracy vs different *β* values of first phase PCA on THCA datasets.

**Fig 5:**
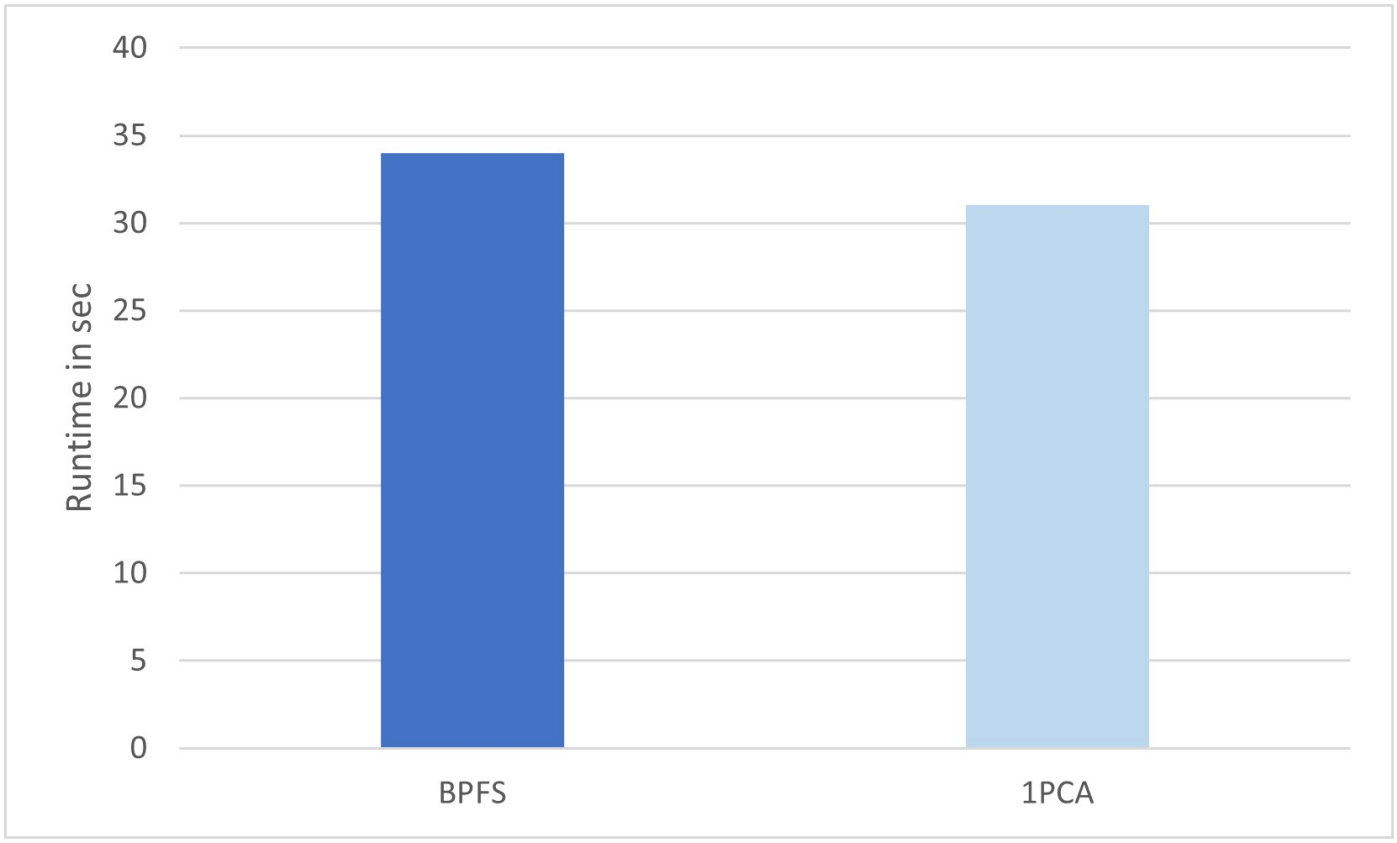
Runtime of the first phase PCA and BPFS on THCA datasets.

As shown in Fig 4, with less genes selected, the classification accuracy on THCA data using the selected gene gets worse, especially when the number of selected gene is less than 73 (i.e., *β* < 0.13%). Comparing to Fig 2, with the same or even less number of selected genes, BPFS can achieve better performance and increase the accuracy nearly 10% when the selected gene number is less than 73. In terms of running time, Fig 5 shows the running time comparison between using first phase PCA only (31 seconds) and BPFS (34 seconds) on THCA dataset. Therefore, the goal of the first phase of PCA is to initially filter the gene domain by choosing the genes that play an important role in the top PCs (by looking at the loading score, i.e., the coefficients), while the second phase of PCA focuses on gene itself, and the goal is to further filter the gene domain by selecting those that can decide the PCs that can cluster genes using the contribution scores calculated from SVD.

In summary, comparing to our counterparts, BPFS achieves the best performance in all six evaluation metrics with double computational speed and less number of selected genes; comparing to the simpler method using only the first phase PCA of BPFS with smaller value of *β* in gene selection, BPFS also outperforms it in terms of classification accuracy with only a few seconds extra, which shows the importance and necessity of the second phase PCA.

### Discussion

The gene subset obtained by our proposed BPFS method can always achieve the best classification result while spending far less runtime, compared to the counterpart methods.

We discuss some conditions for using our BPFS method as below.

- Our proposed method uses classic PCA as fundamental rule for feature selection that may not directly reveal the complex relationship between genes. Our method can be enhanced by incorporating with other forms of PCA, such as Kernal PCA [27] or local PCA.
- In our method, we used contribution scores to filter the data in the second phase in order to further extract important genes and remove redundancy. The contribution score just works as a reference to the importance/contribution of each gene/feature when transforming the basis, it does not necessarily represent the biological correlation between genes and samples.
- Our method is more suitable for the datasets like gene expression data, which suffers from the “curse of dimensionality”, the number of genes is much more than the number of samples. Our method takes the advantage of the small number of samples and PCA to select relevant genes. Our method can perform well if both sample and feature sizes are no more than 60,000.

## Conclusion

Identifying the informative genes and removing the redundancy from the gene expression data is a fundamental task for gene expression data analysis. It has wide applications, such as disease-gene association analysis and gene regulatory network construction. As the gene expression data size getting larger and larger, it becomes more challenging to extract/identify important genes. In this paper, we proposed the BPFS method to select the informative genes, and reduce the feature size and redundancy effectively from the original dataset. The proposed BPFS method overcomes the computational capacity problem of classic PCA in feature selection from gene expression data by adopting PCA first on the samples domain and then on the gene domain for extracting important genes in the process of constructing principal component. We evaluated our method by comparing our BPFS with four state-of-the-art feature selection algorithms on the cancer classification task. The experiments on four cancer gene expression datasets demonstrate the efficiency, accuracy and effectiveness of our proposed method in extracting the informative features and eliminating redundancies.
